# The impact of discrete modes of spinal cord injury on bladder muscle contractility

**DOI:** 10.1186/1471-2490-13-24

**Published:** 2013-05-13

**Authors:** Abhishek Seth, Yeun Goo Chung, Daniel Kim, Aruna Ramachandran, Vivian Cristofaro, Pablo Gomez III, Duong Tu, Lin Huang, Larry I Benowitz, Dolores Di Vizio, Maryrose P Sullivan, Rosalyn M Adam

**Affiliations:** 1 Urological Diseases Research Center, Boston Children’s Hospital, Boston, MA, 02115, USA; 2 Department of Surgery, Harvard Medical School, Boston, MA, 02115, USA; 3 Department of Neurosurgery, Boston Children’s Hospital, Boston, MA, 02115, USA; 4 Division of Urology, Veterans Administration Boston Healthcare System, 1400, V F W Parkway, West Roxbury, MA, 02132, USA; 5 Department of Surgery, Brigham and Women’s Hospital, Boston, MA, 02115, USA; 6 Samuel Oschin Comprehensive Cancer Institute, Cedars-Sinai Medical Center, 8700 Beverly Boulevard, Los Angeles, CA, 90048, USA; 7 Enders Research Laboratories, Rm 1061, 300 Longwood Avenue, Boston, MA, 02115, USA

**Keywords:** Bladder, Smooth muscle, Spinal cord injury, Muscle contractility

## Abstract

**Background:**

Prior studies have compared the effect of spinal cord injury elicited using distinct approaches on motor and visceral function. However, the impact of such discrete modes of injury specifically on bladder muscle contractility has not been explored in detail. The goal of this study is to compare the impact of complete spinal cord transection versus clip compression at thoracic vertebra eight (T8) on bladder muscle contractility.

**Methods:**

Rats underwent no treatment (Control), laminectomy (Sham, SH); complete extradural transection (TX); or cord compression with an aneurysm clip (CX). Bladders and spinal cords were harvested at 6 wk for contractility studies or histological analysis.

**Results:**

Detrusor strips from TX and CX rats showed higher spontaneous activity than those from SH rats. Furthermore, the duration of the neurally-mediated contractile response was longer in TX and CX rats compared to controls and showed attenuated relaxation. No significant differences were observed between muscle strips from SH, TX or CX rats in response to KCl, ATP or phenylephrine. However, tissues from TX and CX rats showed a higher sensitivity to carbachol compared to that from SH animals.

**Conclusions:**

Complete SCI in rats either by cord transection or compression elicits qualitatively similar changes in bladder muscle contractility. Whereas cord transection is arguably easier to perform experimentally, cord compression better models the situation observed clinically, such that each approach has clear advantages and limitations.

## Background

Spinal cord injury (SCI) is a devastating occurrence, affecting up to 12,000 people annually in the United States (http://www.fscip.org/facts.htm). The cost of lifelong treatment for SCI is significant, running into hundreds of thousands of dollars per affected individual [1,2]. Urologic complications account for much of the morbidity associated with SCI and comprise a significant fraction of the associated cost of treatment and rehabilitation. In addition, bladder dysfunction secondary to SCI or congenital neural defects is a source of considerable psychosocial and physical distress to patients [3].

Experimental SCI can be induced in a variety of ways, including crush injury using a clip or Horizon Impactor apparatus [4,5], complete or partial transection of the cord [6-8] or by occluding blood supply to the cord to elicit ischemic injury [9,10]. Previous reports have compared functional outcomes following a variety of complete and incomplete spinal cord injuries in rodents primarily using cord transection or compression to evoke injury [11,12]. Whereas many parameters relevant to voiding such as bladder capacity, voiding efficiency and micturition cycle time were indistinguishable between complete cord transection and cord compression [11,12], selected aspects of lower urinary tract function were different. In particular, the extent of detrusor-sphincter dyssynergia was lower in rats with compression injury compared to those with cord transection [11]. However, the extent to which these differences are maintained at the level of bladder smooth muscle has not been completely defined. In this study we have employed two modes of thoracic spinal cord injury to test the hypothesis that spinal cord injury following complete transection versus clip compression injury elicits distinct effects on bladder smooth muscle contractility. We have chosen thoracic vertebra 8 (T8) as the site of injury as this emulates the phenotype of an upper motor neuron lesion. Injury at this level interrupts all major efferent and afferent pathways including corticospinal, rubrospinal, vestibulospinal, spinothalamic and spinocerebellar tracts. Such injuries are known to elicit a range of consequences including loss of gross and fine motor function, manifest as hindlimb paralysis and loss of proprioception, as well as visceral dysfunction in the gastrointestinal and urinary tracts. In particular, suprasacral injury is effective in eliciting a phenotype comprising an overactive/reflex bladder.

## Methods

### Creation of spinal cord injury in rats

Thirty three male Sprague–Dawley rats (6 wk of age) were divided into 3 groups of 11: sham-operated rats (SH, laminectomy only); transected rats (TX, complete extradural cord transection at T8); and clip compression rats (CX, cord compression at T8). A fourth cohort of rats received no treatment (Control). Injury at T8 models an upper motor neuron lesion and elicits a reflex bladder phenotype with overactivity. Under general anesthesia induced with ketamine (75 mg/kg, i.p.) and medetomidine (0.5 mg/kg, i.p.), a dorsal midline incision was made over the thoracic spinal cord. Superficial and deep muscle layers were incised in the midline to expose the spine. Sham operated animals underwent laminectomy only, while TX rats received a complete extradural transection at T8. A straight microaneurysm clip (0.8 mm × 5 mm) imparting 60 g closing force (Harvard Apparatus, Harvard, MA) was used to compress the cord at T8 for 60 sec in CX rats. Post-operative pain was managed with meloxicam (1 mg/kg, i.p.) analgesia. During the period of spinal shock, which lasted from 1-3 wk following creation of SCI, bladders of rats were emptied twice daily by manual compression with care taken to avoid unintentional bladder rupture. Six weeks following creation of SCI, bladders were harvested for endpoint evaluation. All bladders were weighed and either placed in Hank’s balanced salt solution for 15 min prior to fixation in neutral-buffered formalin for histological analysis, embedded in O.C.T for molecular evaluation or processed for contractility testing as outlined below. All animal studies were approved by the Boston Children’s Hospital Animal Care and Use Committee prior to experimentation.

### Ex-vivo contractility assay

At 6 wk after SCI, bladders from 5 rats in each cohort were harvested and preserved in ice-cold Kreb’s buffer (NaCl 120 mM; KCl 5.9 mM; NaHCO_3_ 25 mM; Na_2_H_2_PO_4_ 1.2 mM; MgCl • 6H_2_O 1.2 mM; CaCl_2_ 2.5 mM; dextrose 11.5 mM) for *ex vivo* contractility analyses. Bladder tissue was carefully cut into strips and the mucosa dissected off the detrusor muscle under microscopic guidance. Detrusor strips were suspended in an organ bath maintained at 37°C and bubbled with a mixture of 95% O_2_ and 5% CO_2_. Tissues were attached to a force transducer (Grass Instruments), stretched to a resting tension of 1.5 g and equilibrated for 45 min. Contractile responses to phenylephrine (adrenergic agonist, 100 μM), carbachol (cholinergic agonist, 1 nM-10 μM), α,β-methylene-ATP (purinergic agonist, 10 μM), KCl (120 mM), and to electrical field stimulation (1-64 Hz, 20 V, 0.5 ms pulse width, 10 sec duration) were measured in separate strips. Conditioned signals from force transducers were continuously acquired at 30 Hz by a 16-channel analog-to-digital converter (DataQ, DI-720) and recorded to disk using Windaq data acquisition software. Data were expressed as force (mN) normalized by tissue cross-sectional area and presented as mean ± SEM. The area under the curve of EFS-induced contractions was calculated at each frequency as the integral of force over the time interval from the beginning of stimulation to the return to 10% of the maximum contraction.

Characterizing the pattern of spontaneous activity by conventional methods that quantify the amplitude and frequency of oscillations can be challenging, particularly after SCI. Therefore, spontaneous activity of muscle strips was assessed in the frequency domain by discrete Fourier transformation (DFT), as described [13]. A time series of 4.5 min (8191 data points) was selected for analysis from data acquired under resting conditions prior to the stimulation protocols above. The power spectrum generated from the DFT was smoothed using a moving average filter (Windaq waveform analysis software). The power spectrum describes the strength of oscillations as a function of frequency and peaks in the spectra identify the predominant frequencies composing the spontaneous activity signal. These peaks were detected as local maxima and the corresponding frequency at which the peaks occurred was recorded for each tissue strip. Power was expressed in decibels as the log transformed relative magnitude of spontaneous oscillations.

### Catheter placement and awake unrestrained cystometry

Five wk after surgery, a suprapubic (SP) catheter was inserted in up to 8 rats in each cohort and tunneled under the skin to the back of the neck. Cystometry was conducted 1-3 d after SP catheter placement. The SP catheter was attached to a physiological pressure transducer (MLT844 AD Instruments) to allow measurement of intravesical pressure, while bladder was continuously infused with sterile PBS at 100 μl/min. Pressure readings were converted to digital signals using a PowerLab data acquisition system and analyzed using LabChart Pro software (ADInstruments, Colorado Springs, CO). Post void residual volumes were measured by aspirating the SP catheter at the conclusion of cystometry. A spontaneous non-voiding contraction (SNVC) was defined as any rise in intravesical pressure of greater than 5 cm H_2_O.

### Statistical analysis

Analysis of contractility data was conducted using analysis of variance (ANOVA) followed by Holm-Sidak or Dunn’s post-hoc analysis for normally and non-normally distributed data respectively. Normality was determined by Shapiro-Wilk tests. SigmaStat was used to carry out statistical analyses.

## Results

### Histologic characterization of spinal cords and bladders in injured rats

**Figure 1:**
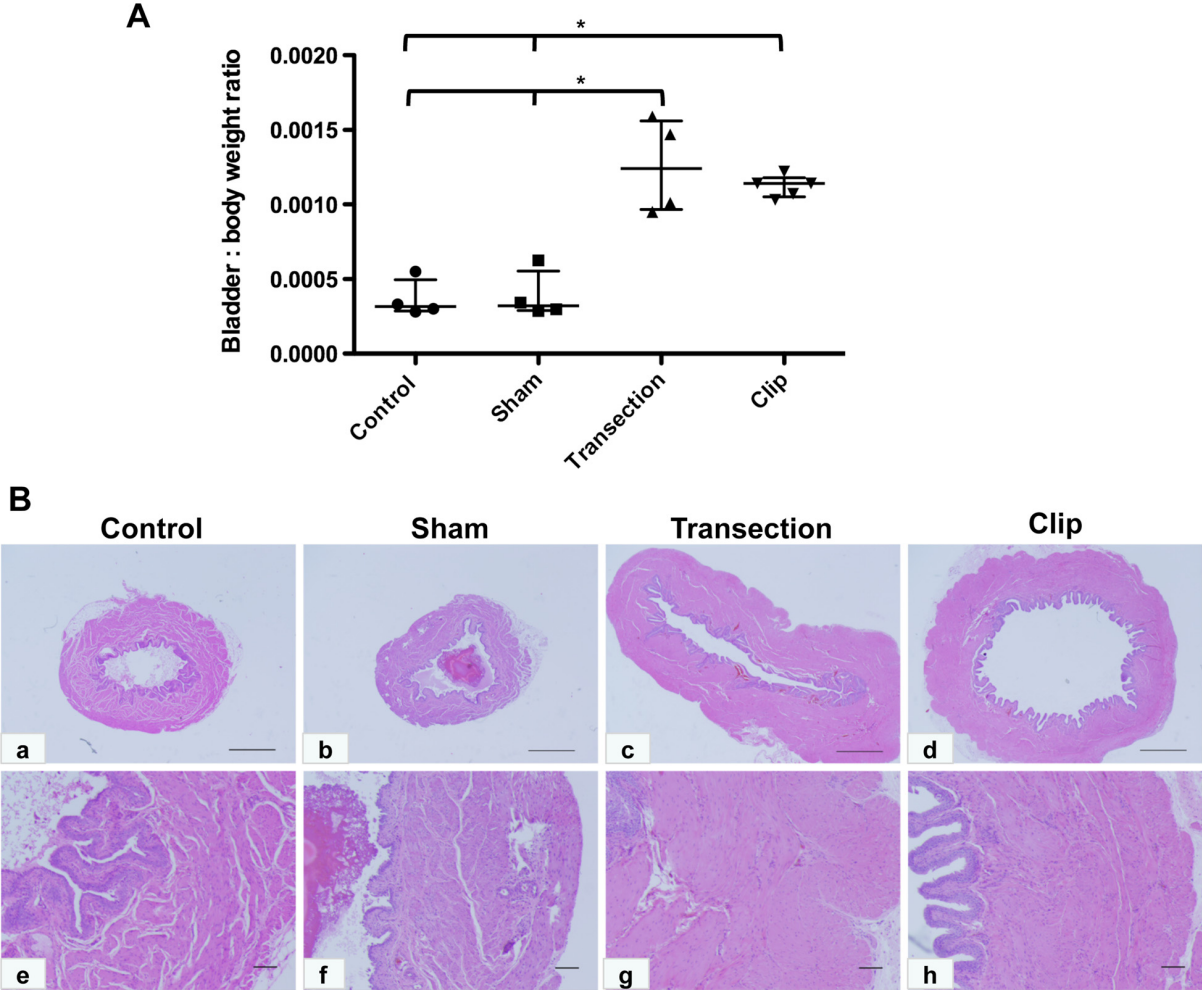
**Comparable remodeling of the bladder wall in spinal cord injured rats compared to controls.** (**A**) Bladder-to-body weight ratios were calculated from non-operated controls (n = 4), laminectomy (Sham, n = 4), spinal cord transection (Transection, n = 4) or spinal cord clip compression (Clip, n = 5) groups. (**B**) H&E-stained bladder wall sections at low (a-d) and high (e-h) magnification are shown. Scale bars: a-d, 100 μm; e-h, 10 μm.

**Figure 2:**
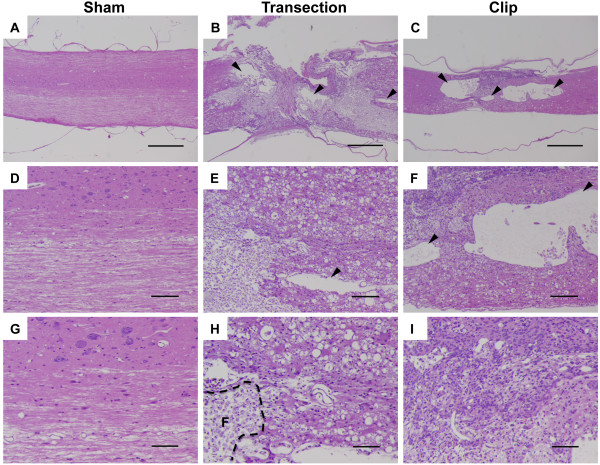
**Distinct histological changes in spinal cord sections from sham and injured rats.** Spinal cords from sham (**A**, **D**, **G**), transected (**B**, **E**, **H**) or clip compression (**C**, **F**, **I**) rats were harvested at 6 wk post injury and stained with H&E. Scale bars = 5 mm (**A**, **B**, **C**); 100 μm (**D**, **E**, **F**); 20 μm (**G**, **H**, **I**). Arrowheads in (**C**) indicate pseudocysts that form within the cord following injury. The dotted line in (**H**) indicates an area enriched with foamy cells (**F**).

To compare the nature and extent of injury in rats exposed to different types of spinal insult, we performed histologic analysis of bladders and spinal cords from each cohort of rats. Consistent with previous reports in the literature [14], the bladder-to-body weight ratios in TX and CX rats were significantly higher than in SH animals, as a result of increased bladder weights following SCI (*, p < 0.05 in each case, Figure 1A). Compared to bladders of sham-operated rats that received laminectomy only, bladders from animals with compression or transection injury showed a marked increase in size (Figure 1B). Although specimens from rats with transection injury showed a greater variation in bladder-to-body weight ratio than those from rats with compression injury, there was no significant difference in bladder wall appearance in each case. In contrast to cords from SH animals, sagittal sections of cords obtained from TX and CX rats displayed variable amounts of foamy cells occupying areas of cavitation (Figure 2H). The cavities were frequently larger and completely formed in CX rats and displayed features of pseudocysts (Figure 2C, 2F). In contrast, cavities were more disorganized and smaller in the TX rats and were more frequently surrounded by a glial scar. The presence of edema, necrosis, and fibrosis appeared to correlate with the extent of the injury. Neuronal damage, consisting of neuronal swelling and chromatin dispersion with focal chromatolysis, was particularly severe in close proximity to the lesion in both TX and CX rats.

### Voiding behavior in spinalized rats

Routine evaluation of rats revealed a return of voiding activity 2–3 wk after injury, indicating the end of the spinal shock period. Consistent with previous reports, voided volumes in SCI rats were larger than those in SH controls following injury. At wk 3, mean voided volumes in TX and CX were 2.63 ± 0.84 ml and 2.50 ± 0.86 ml respectively, which were significantly higher than mean voided volume in SH of 1.40 ± 0.64 ml; at wk 5, mean voided volumes in TX and CX were 2.76 ± 0.86 ml and 2.27 ± 0.61 ml, respectively compared to 1.32 ± 0.67 ml in SH rats. At each time point, differences in voided volumes between spinal cord injured animals and sham rats were statistically significant (TX vs SH, p < 0.05; CX vs SH, p < 0.05). However, no significant difference was observed in voided volumes between TX and CX rats.

Cystometric analyses in conscious animals revealed variable degrees of bladder overactivity in both TX and CX rats, that was not observed in SH animals. Spontaneous non-voiding contractions (SNVCs) were seen consistently during each filling phase in 7 of 8 TX rats, 6 of 7 CX rats, but in 0 of 7 SH rats. The mean number of SNVCs per voiding cycle in the TX and CX groups were 3.6 and 3.9 respectively, compared to SH rats, which had a mean SNVC frequency of <1 per voiding cycle. Although spinalized rats had significantly greater overactivity compared to controls, no difference in this parameter was observed between TX and CX animals.

### Ex vivo contractility analysis

**Figure 3:**
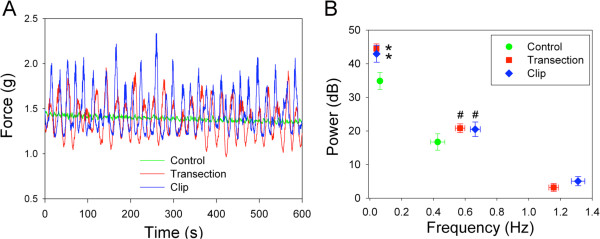
**Increased spontaneous activity in bladder strips from injured rats versus controls.** (**A**) Representative tracings of spontaneous activity in bladder tissue from control, TX and CX rats. (**B**) Comparison of relative magnitudes at corresponding frequencies determined from power spectra of spontaneous activity in each group (*significantly higher power and lower frequency than control, p < 0.05; # significantly higher frequency than control, p < 0.05).

To investigate potential mechanisms underlying the altered voiding behavior observed following SCI, we performed contractility analyses on isolated bladder muscle strips. We observed significantly higher amplitude spontaneous activity in bladder tissue from both TX and CX rats compared to SH controls. The irregular amplitude and frequency of spontaneous activity observed in SH rats contrasted sharply with the uniform spontaneous oscillations in injured animals that were more organized and complex. This altered pattern of activity is illustrated in Figure 3, which shows low frequency, high power events in all groups; however, the power spectral peak was significantly higher and the frequency was lower in the injured groups (p < 0.05). The mid-frequency component of spontaneous activity was shifted to significantly higher frequencies in injured animals, although the power spectral peaks at these frequencies were not different from non-injured animals. Interestingly, a high frequency/low power component of spontaneous activity emerged only in tissues from injured animals (Figure 3B).

**Figure 4:**
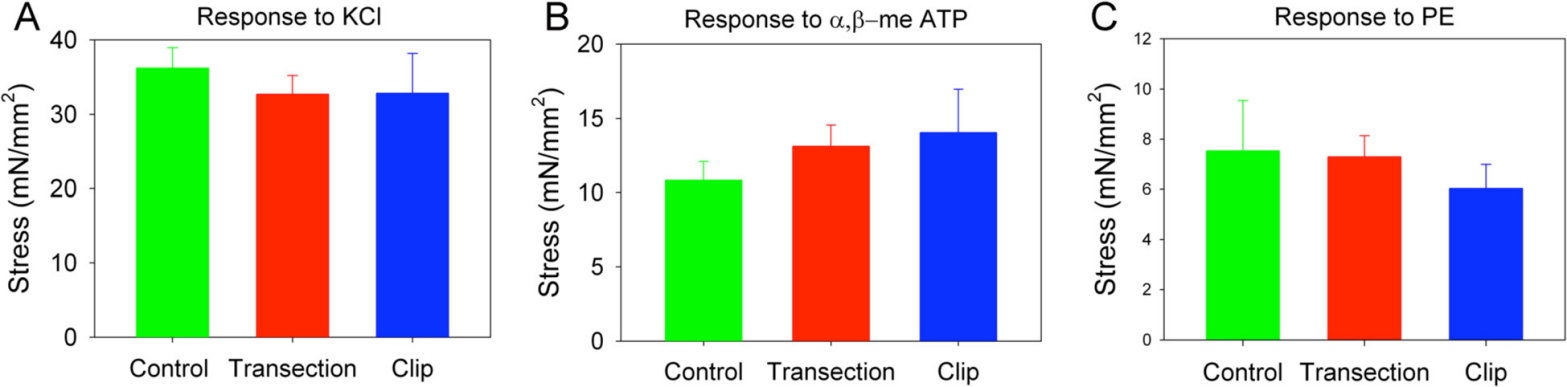
**Comparable ex vivo contractility of detrusor tissue strips from rats with transection versus compression injury.** Contractile responses to (**A**) increased extracellular KCl (120 mM), (**B**) α-β-me-ATP (10 μM) and (**C**) phenylephrine (100 μM) in control, TX and CX bladders.

**Figure 5:**
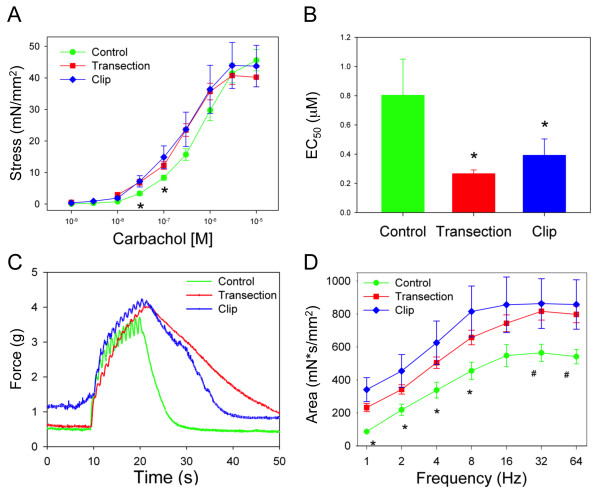
**Enhanced cholinergic sensitivity in detrusor tissue strips from injured rats versus controls.** (**A**) Dose–response curves for carbachol in control, TX and CX bladders. *, control significantly lower than TX and CX, p < 0.05. (**B**) EC_50_ values generated from concentration-dependent carbachol contractions in bladder tissue from control, TX and CX. *, significantly lower than control, p < 0.05. (**C**) Original representative tracings from each group showing contractile responses to EFS at a frequency of 1 Hz. Stimulation began at 10 sec and terminated at 20 sec. Data represent mean ± SEM. (**D**) Frequency-response curves for EFS expressed as area of contraction. *, significantly lower than TX and CX, p < 0.05; #, significantly lower than TX, p < 0.05.

Upon evaluation of agonist-induced alterations in muscle contractility, there was no significant difference noted in tension generation in response to KCl, α,β-me-ATP or phenylephrine (PE) between tissues from SH, TX or CX rats (Figure 4A - C). However, muscle strips from spinalized rats were more sensitive to carbachol, a cholinergic agonist and parasympathomimetic agent (Figure 5A). The half maximal effective concentration (EC_50_) of carbachol was significantly lower in muscle strips from injured rats than in control rats (p < 0.05, Figure 5B). Although the contractile response was significantly lower in control animals at low carbachol concentrations, the maximum response was not different among groups.

The frequency-response curves generated by EFS in both SCI groups were not different from controls. While the maximum amplitude of contraction was similar among groups, the time course of the contractile response was markedly altered in injured animals, with prolonged duration of contraction and attenuated relaxation (Figure 5C). Consequently, the area of the EFS-induced responses was greater in TX and CX rats, particularly at lower frequencies of stimulation (Figure 5D).

## Discussion

Previous reports comparing the effect of complete and incomplete SCI by cord transection or compression, respectively, on lower tract function have yielded somewhat different results [11,12]. Using a weight drop contusion model of SCI versus complete cord transection, Pikov and colleagues demonstrated some restoration of coordinated voiding behavior within 2 wk of injury in rats with cord contusion, that was not observed in rats with cord transection [11]. In a related study, larger expressed bladder volumes were noted in rats with spinal cord transection, compared to animals with incomplete cord injury [12], suggesting that injury severity is a key variable in dictating restoration of function. In our study, both spinal cord compression and transection evoked complete injury, in agreement with the comparable functional outcomes in tissue remodeling, voiding behavior and muscle strip contractility.

In organ bath studies, we observed significantly higher spontaneous activity of bladder muscle strips from injured rats compared to controls. Previous studies suggest that SCI causes the re-emergence of a neonatal pattern of spontaneous activity [15]. During early postnatal development, large amplitude/low frequency activity has been described [16], similar to the activity observed in our injured animals. Developmental changes examined by Fourier analysis previously demonstrated a low frequency component (0.08-0.21 Hz) that emerged after a few weeks of age and a second component (~0.5 Hz) that arose after 3 wk of age. After 5 wk of age, the magnitude of the low frequency component was reduced while the second component was predominant. These data are consistent with the frequency spectra that we detected in adult control animals and the shift to higher magnitude at low frequency after injury corresponding to the postnatal pattern. However, the complexity in the pattern of activity was greater in SCI, as evidenced by higher frequency components in the power spectra that became apparent after SCI but were absent in non-injured and neonatal animals [16].

Among the stimulus-evoked responses in muscle strips, significant differences were observed in the SCI groups in response to EFS and carbachol. An increase in acetylcholine release from nerve terminals has been previously reported after SCI due to a shift from pre-junctional M1 muscarinic receptor-mediated facilitation of neurotransmission to high affinity M3 receptor activated release of acetylcholine [17]. This change in pre-junctional regulation of cholinergic neurotransmission may underlie the augmented duration of the contractile response to EFS in TX and CX rats. The increased sensitivity of muscle strips from SCI rats to carbachol, without a change in the contractile response to KCl, suggests dysregulation of post-synaptic muscarinic receptor signaling. Although cholinergic contractile responses are normally mediated by the M3 muscarinic receptor subtype, alterations in M2 receptor signaling in smooth muscle have been reported post-SCI. In the rat, SCI enhanced the response to a M2 receptor agonist and increased M2 receptor gene expression [18], suggesting an augmented contribution from M2 receptor signaling to cholinergic contraction in SCI bladders. A similar shift from M3 to M2 receptor mediated contraction has been described in patients with neurogenic bladder [19].

## Conclusions

In summary, both transection and clip compression models of SCI provide similar, reproducible changes in several parameters relevant to lower urinary tract function. Whereas cord transection is arguably easier to perform experimentally, cord compression better models the situation observed clinically, such that each approach has clear advantages and limitations. These studies provide a basis for subsequent investigations into agents that may target the deleterious consequences of SCI.

## Abbreviations

ANOVA: Analysis of variance; CX: Spinal cord compression; EFS: Electrical field stimulation; i p: Intraperitoneal; KCl: Potassium chloride; O C T: Optimal cutting temperature compound; SCI: Spinal cord injury; SH: Sham-operated; TX: Spinal cord transection.

## Competing interests

The authors declare they have no competing interests.

## Authors’ contributions

AS, MPS and RMA conceived of the study; AS, YGC, DK, AR, PG and DT performed spinal cord surgeries, post-operative management of rats, tissue harvesting and preparation of tissue for histological analysis; LH, VC and MPS performed statistical analysis; LIB consulted on creation of spinal cord injury; DDV performed histopathological analysis of tissues; AS, MPS and RMA wrote the manuscript. All authors read and approved of the final manuscript.

## Pre-publication history

The pre-publication history for this paper can be accessed here:

http://www.biomedcentral.com/1471-2490/13/24/prepub
